# Acute Exertional Lateral Compartment Syndrome: A Diagnostic Challenge Presenting With Peroneal Nerve Palsy and Myoglobin‐Mediated Acute Kidney Injury—A Case Report

**DOI:** 10.1155/cro/5885194

**Published:** 2026-08-31

**Authors:** Marlon M. Mencia, Cameron J. Mencia, Pedro Pablo Hernandez-Cruz

**Affiliations:** ^1^ Department of Clinical Surgical Sciences, University of the West Indies, St. Augustine, Trinidad and Tobago, uwi.edu; ^2^ Department of Surgery, Port of Spain General Hospital, Port of Spain, West Indies, Trinidad and Tobago; ^3^ Imperial College London, London, UK, imperial.ac.uk

**Keywords:** acute kidney injury, case report, compartment syndrome, exercise, fasciotomy, peroneal nerve

## Abstract

**Background:**

Acute exertional compartment syndrome (AECS) is a rare but limb‐threatening condition that typically occurs in athletes or military recruits in the absence of trauma. Although the anterior compartment of the leg is most commonly affected, isolated lateral compartment involvement is distinctly uncommon. Delayed recognition is frequent and may result in serious complications, including acute kidney injury (AKI) and amputation.

**Case Presentation:**

We report the case of a 37‐year‐old previously healthy man who presented with severe lateral leg pain, progressive swelling and acute foot drop approximately 12 h after a high‐intensity interval training (HIIT) session. Initial investigations revealed markedly elevated creatine phosphokinase, myoglobin and creatinine levels, and he was admitted with a provisional diagnosis of rhabdomyolysis‐induced AKI. Despite aggressive intravenous hydration, his biochemical parameters worsened and leg pain intensified. Clinical assessment demonstrated a tense lateral compartment and a diagnosis of acute exertional lateral compartment syndrome was made, and urgent fasciotomy was performed. Postoperatively, pain rapidly improved and renal function normalised over several days. At 2‐year follow‐up, the patient had residual weakness of ankle dorsiflexion (MRC Grade 4) but had returned to moderate physical activity.

**Conclusion:**

Acute exertional lateral compartment syndrome is an exceptionally rare diagnosis and may present atypically, leading to delayed treatment. Clinicians should maintain a high index of suspicion in patients presenting with severe leg pain and neurological deficit following exercise. Early diagnosis and prompt fasciotomy remain critical to optimising functional outcomes.

## 1. Introduction

Richard von Volkmann is widely credited with the first description of acute compartment syndrome (ACS) in 1881; however, this attribution has recently been questioned [1, 2]. Aslanabadi et al. identified a description consistent with ACS in the English translation of a medical treatise by Abdul Qasim Al‐Zahrawi, written in Córdoba, Spain, around 980 AD [1]. In this account, Al‐Zahrawi described the progressive deterioration of a young boy following an open fracture of the leg, recognising the consequences of impaired blood flow and muscle injury. This observation may represent the earliest recorded description of ACS. Untreated ACS results in irreversible muscle and nerve ischaemia, causing permanent loss of function if not recognised and treated promptly [3–6]. Although ACS is well known to all medical students, its close relative, acute exertional compartment syndrome (AECS), remains relatively unrecognised, with fewer than 100 published cases [3, 7–13]. Like ACS, which typically occurs after injury, AECS is a surgical emergency requiring urgent fasciotomy to prevent permanent neurovascular damage [4]. This peculiar variant was described by P.R. Vogt as ischaemic muscular necrosis in his presentation at the Oregon State Medical Society Meeting in 1943, and later published by E. Severin in the same year [14].

Most cases of AECS occur in the lower leg, particularly in the anterior compartment, although AECS in other compartments has also been documented [9–12, 15]. The first recorded cases of acute lateral compartment syndrome following exertion were described in a seminal 1957 paper by Blandy and Fuller [14]. Timely and accurate diagnosis of AECS is crucial, as any delay increases the risk of complications. Despite nearly 80 years since the initial descriptions, AECS remains poorly understood and is frequently misdiagnosed. In this report, we present a rare case of AECS of the lateral compartment, which was initially misdiagnosed resulting in rhabdomyolysis and acute kidney injury (AKI).

## 2. Case Presentation

A 37‐year‐old man presented to our Emergency Department at 2 a.m., complaining of severe pain in his right calf and loss of ankle movement. The pain began 12 h earlier, following a 45‐min high‐intensity interval training (HIIT) session. Table 1. At the end of the exercise session, the patient recalled a feeling of tightness in his calf, similar to but less intense than a previous episode that had occurred a month earlier whilst on a 4‐day hiking trek. Whilst driving home in heavy traffic, his calf became painful, and he was unable to lift his ankle to safely operate the foot pedals. By the time he arrived home, he was unable to walk and required assistance to get into his flat. Over the next few hours, the pain worsened, and he began vomiting, prompting him to seek medical attention.

**Table 1:** Patient′s high‐intensity interval training regimen prior to presentation.

Exercise	Sets	Reps
Jump squats	3	10
Push‐ups	3	15
Mountain climbers	3	10
Burpees	5	10
High knees/sprint in place	3	1 min
Kettlebell swings	5	15
Walking lunges	3	10 each side
Jump squats	3	5

On presentation, the patient′s leg was swollen and tender, with an obvious foot drop. Although radiographs of the leg were normal, laboratory tests showed elevated levels of creatine phosphokinase (CPK), myoglobin and creatinine. The patient was admitted to internal medicine with a working diagnosis of AKI secondary to rhabdomyolysis and started on intravenous fluid therapy. Despite treatment, his blood indices continued to rise, and he was referred to nephrology. In the face of deteriorating renal function and worsening leg pain, an orthopaedic consultation was also requested.

The patient was seen by the orthopaedic team 18 h after admission and during the initial assessment, the team noted moderate swelling and erythema on the lateral aspect of the leg just below the fibular head. The area was firm and exquisitely tender, and the patient had a dense common peroneal nerve palsy with normal pedal pulses. Based on these findings, we diagnosed ACS and immediately prepared the patient for fasciotomy.

**Figure 1:**
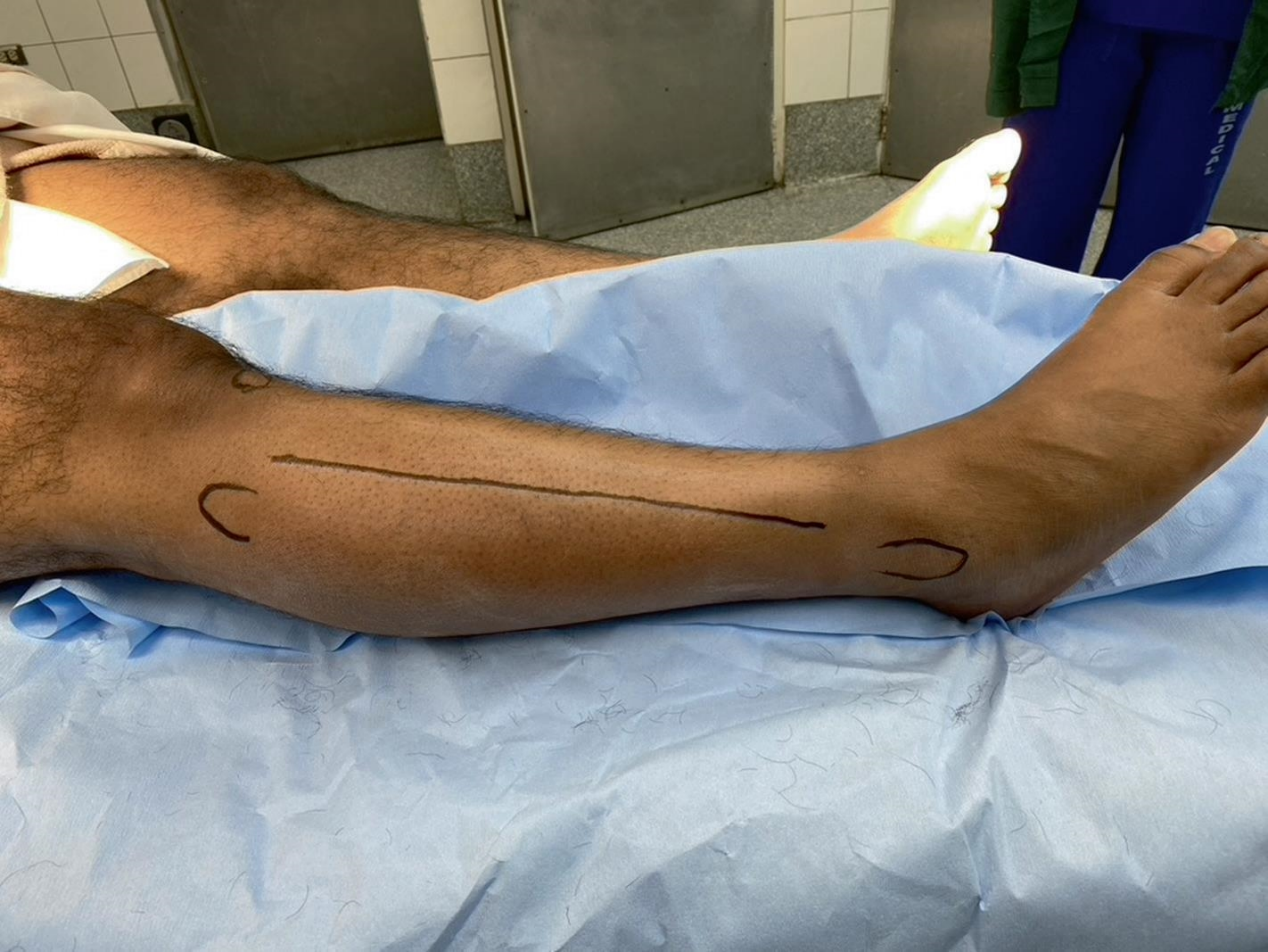
Preoperative clinical picture showing the fibular head, lateral malleolus and tibial tuberosity as reference points. Note the marking for the skin incision (solid line), which is made one finger′s width anterior to the edge of the fibula.

**Figure 2:**
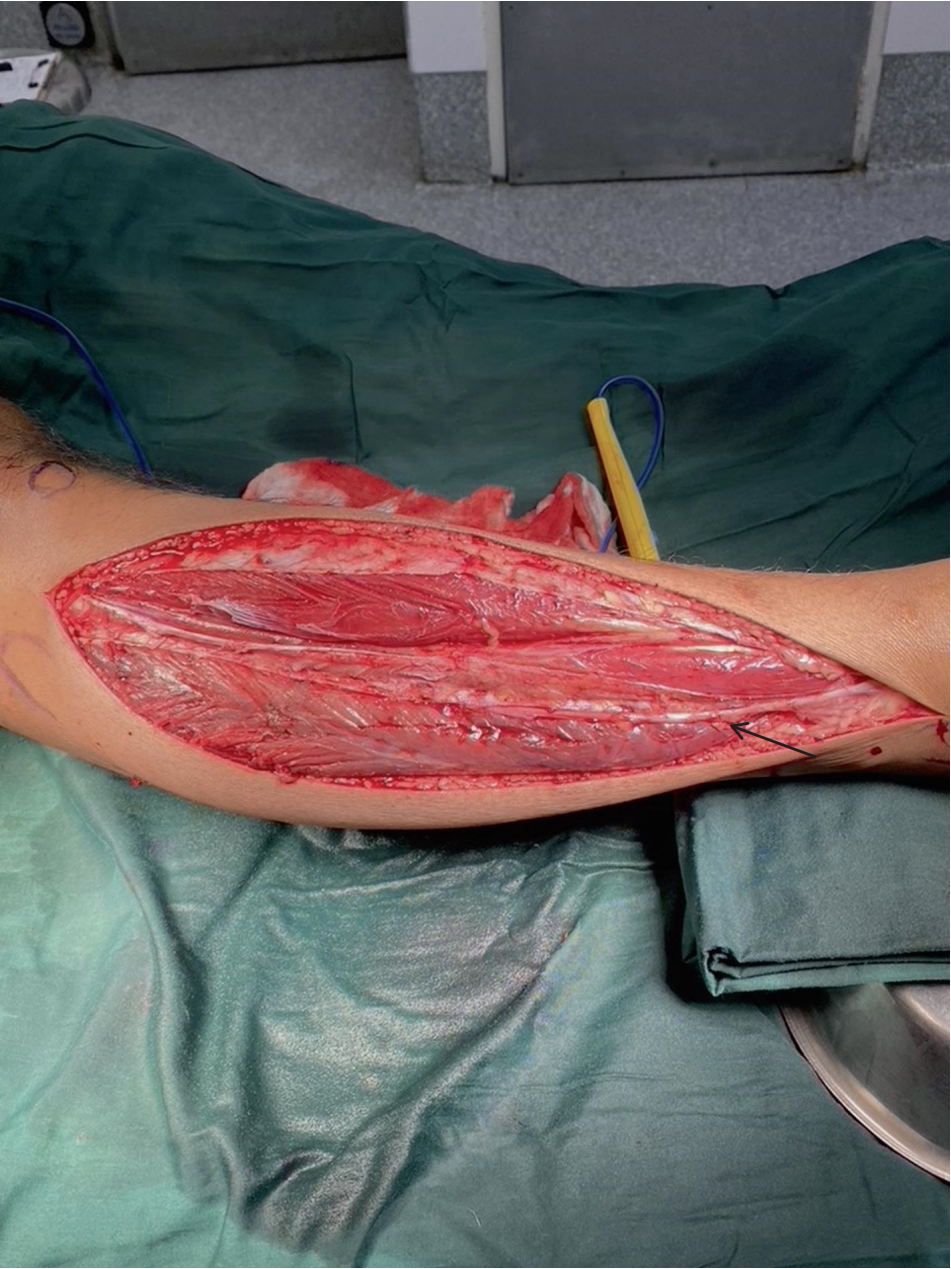
Intraoperative photograph of the leg. Complete fasciotomy of the anterior and peroneal compartments. Note the superficial branch of the peroneal nerve (black arrow) has been preserved.

At surgery, the lateral compartment was approached via a 20‐cm incision, one finger′s width anterior to the fibula, extending distally from the fibular head to the lateral malleolus, which permitted complete release of the lateral compartment in accordance with Bowyer′s recommended technique [16] (Figure 1). We identified the intermuscular septum and released the fascia overlying the muscles using the classic ‘H’‐shaped incision, whilst protecting the terminal branches of the peroneal nerve [16]. Upon release, the swollen muscles immediately bulged through the fascia, confirming that the compartment pressure was elevated (Figure 2). The proximal muscle appeared dusky, but there was no necrotic tissue, and the muscle belly contracted briskly to stimulation, with bright red capillary bleeding.

**Figure 3:**
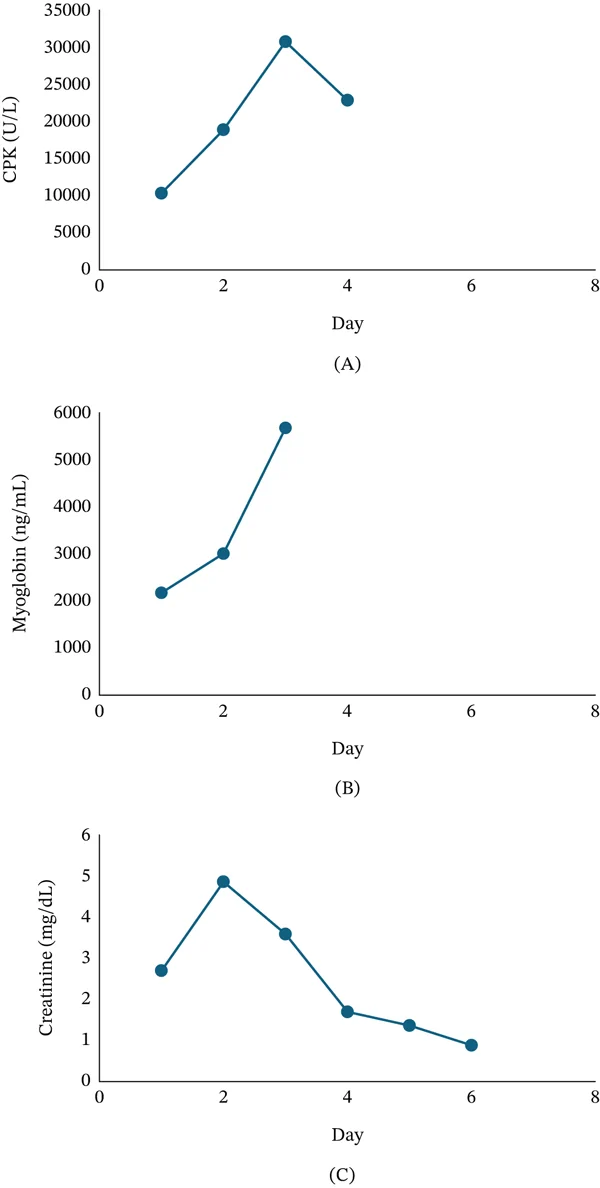
Serial changes in (A) creatine phosphokinase, (B) myoglobin and (C) creatinine during the acute clinical course.

**Figure 4:**
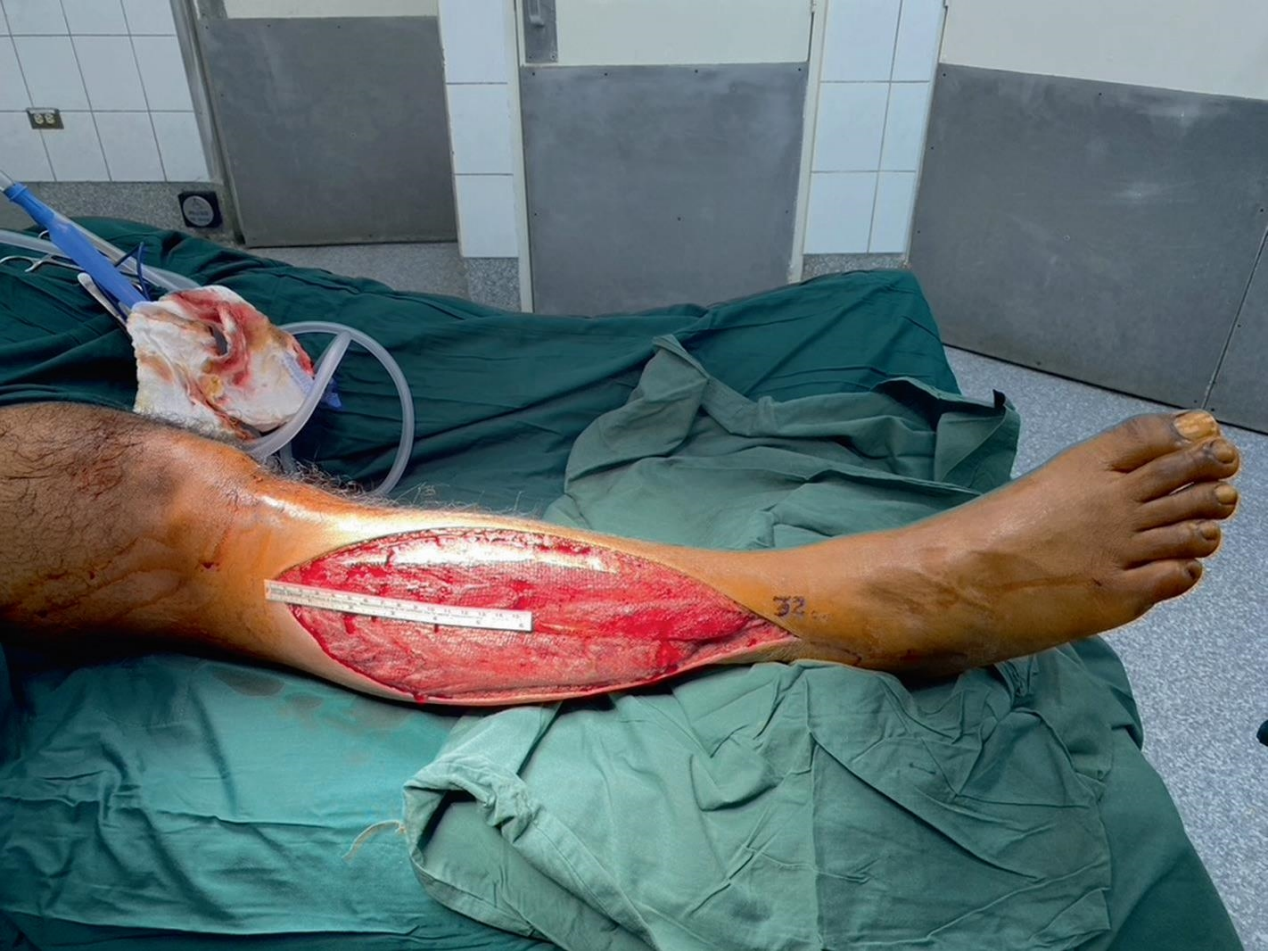
Intraoperative photograph taken prior to skin closure using vascular loops. The wound measures 32 × 11 cm. Exposed surface area is 276 cm^2^.

**Figure 5:**
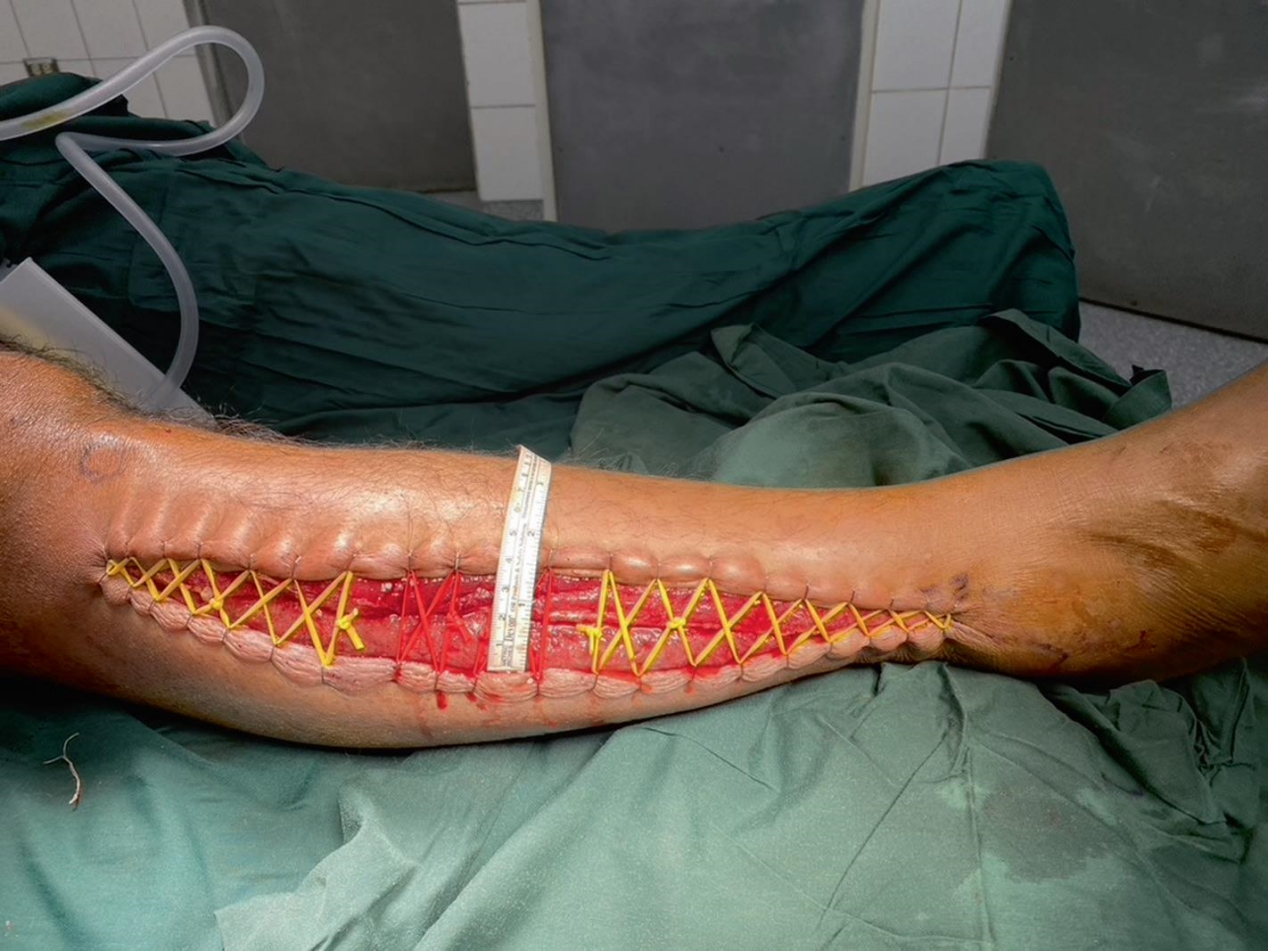
Intraoperative photograph taken after skin closure using vascular loops. The wound measures 32 × 3.5 cm. Exposed surface area is 88 cm^2^ (68% reduction in size).

**Figure 6:**
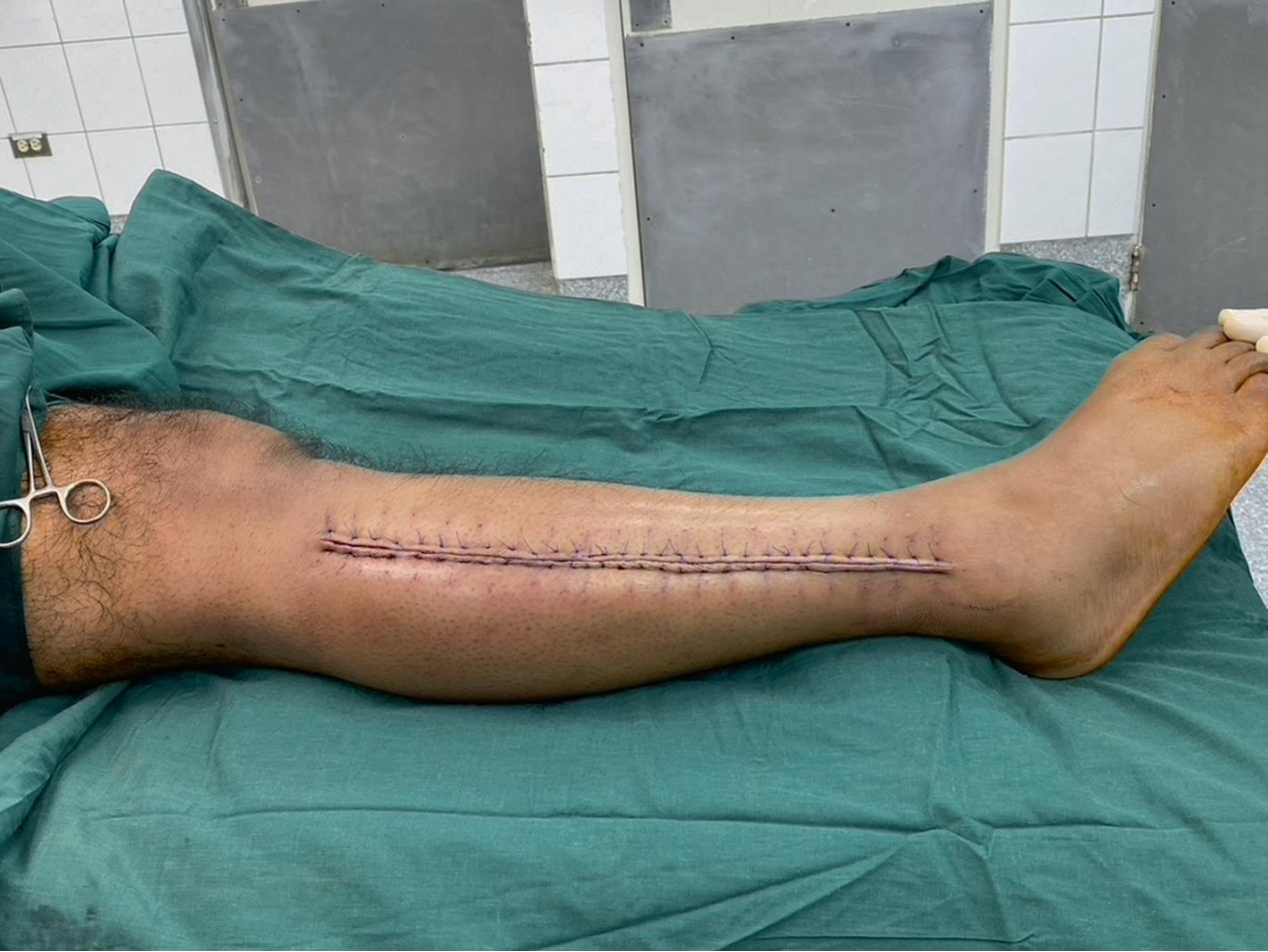
Intraoperative photograph showing final skin closure.

After confirming complete release of the lateral compartment, we then turned our attention to the posterior compartment but the calf muscles had remained soft throughout so we decided not to decompress the posterior compartment and avoid the morbidity of a second incision. Finally, the wound was heavily bandaged, and a foot‐drop splint was applied. The following morning, the patient reported only mild discomfort, and CPK and creatinine levels rapidly normalised 5 days after admission (Figure 3 A–C). The patient returned to the operating theatre on two further occasions, where skin closure was achieved using the shoelace technique and a horizontal mattress suture, which provided robust dermal purchase and even distribution of wound tension [17, 18] (Figures 4, 5 and 6). Two weeks following hospital discharge, the incision was fully healed, but his peroneal nerve function had not recovered. He was advised to use a foot‐drop splint and referred for physiotherapy.

At the final follow‐up 2 years after the incident, the patient had reduced ankle dorsiflexion strength (MRC Grade 4) but continued to engage in moderate physical activity. The patient gave informed consent for the anonymized use of his data in this manuscript.

## 3. Discussion

ACS is most commonly associated with trauma, typically fractures, and is considered a surgical emergency that demands prompt fasciotomy to prevent irreversible muscle and nerve damage. Similarly, AECS, which usually occurs without injury but after prolonged physical exercise, is treated by urgent fasciotomy [4]. Regrettably, however, clinicians are largely unfamiliar with the presentation of AECS, leading to delayed or incorrect diagnosis [4, 7, 19]. In a study by Livingston et al., which compared AECS with fracture‐related ACS, four out of seven patients with AECS were misdiagnosed on admission, and the average time to fasciotomy was 97 h, compared with 19 h in the fracture‐related group. Two patients who were misdiagnosed had significant neurological and functional deficits at final follow‐up [3].

Acute muscle hypertrophy, which can increase volume by up to 20%, and increased capillary permeability are primary causes of elevated intracompartmental pressures during exercise [15, 20]. However, anatomical factors are thought to partly explain why certain muscle compartments are more susceptible to exertional compartment syndrome. The anterior compartment of the lower leg is particularly vulnerable to disturbances in blood flow and high pressures that lead to muscle ischaemia following exercise. Ruggeri postulated that anatomical narrowing of the middle one‐third of the anterior compartment results in higher pressures in that area, even with an equal increase in volume throughout the muscle [21]. In the largest retrospective review of AECS, Reneman found that 43 out of 52 cases were confined to the anterior compartment, six were isolated to the lateral compartment, and three involved a combination of the anterior and lateral compartments [15]. Unlike traumatic compartment syndrome, which may involve multiple compartments Reneman reported isolated lateral compartment involvement in six patients with AECS supporting a selective decompression strategy when clinical findings clearly localise the pathology. Furthermore, our patient′s presentation of atraumatic lateral‐sided leg pain likely contributed to a delay in both diagnosis and treatment.

In cases of lateral compartment syndrome, clinicians may reasonably expect motor weakness to result from direct muscle damage and/or injury to the superficial peroneal nerve. Reneman′s analysis of exertional compartment syndrome of the leg found that although most patients presented with muscle paralysis, at surgery only patchy areas of muscle necrosis and fibrosis were observed. He therefore concluded that nerve injury, rather than muscle damage, resulted in the loss of active movement [15]. This condition involves the deep peroneal nerve in lateral compartment syndrome and was first documented by Hiramatsu et al. [22]. The researchers hypothesised that the nerve is vulnerable to compression by the adjacent peroneus longus during its course within the fibular tunnel [23, 24]. Consequently, lateral compartment syndrome may lead to paralysis of the muscles innervated by both the superficial and deep peroneal nerves. Thus, the absence of characteristic clinical symptoms can delay recognition of lateral compartment syndrome. Clinicians must therefore be aware that complete foot drop (involving the superficial and deep peroneal nerves) does not exclude a diagnosis of lateral compartment syndrome.

Rhabdomyolysis results from muscle damage that releases intracellular components, such as creatine kinase and myoglobin, into the extracellular fluid and circulation [25, 26]. Trauma is the leading cause, but excessive metabolic demand during exercise can also lead to exertional rhabdomyolysis [27]. Furthermore, rhabdomyolysis can result in compartment syndrome, which exacerbates muscle damage and ultimately precipitates AKI [28, 29]. Diagnosing exertional rhabdomyolysis can be challenging because creatinine levels naturally rise after exercise, and there is considerable individual variability in response to the same exercise dose [30]. Environmental factors, including high humidity and ambient temperatures, have been shown to increase the risk of rhabdomyolysis [31]. However, the incidence of exertional rhabdomyolysis amongst active young people is generally low [32].

On the day prior to hospital admission, the patient engaged in a 45‐min HIIT exercise session on an unusually hot mid‐December afternoon, with temperatures ranging from 74°F to 85°F and a humidity of 82%. We hypothesise that the prevailing climatic conditions, together with the intense exercise, provoked rhabdomyolysis in our patient, which in turn precipitated lateral compartment syndrome and ultimately AKI.

## 4. Conclusions

Acute exertional lateral compartment syndrome is an extremely rare diagnosis, but should be considered in any patient with a history of recent exercise who presents with increasing pain, a tense peroneal compartment and loss of ankle movement. Delays in treatment may lead to severe consequences and long‐term disability. Clinicians must maintain a high index of suspicion, as early diagnosis and prompt fasciotomy are key to avoiding complications.

## Funding

No funding was received for this manuscript.

## Disclosure

The authors have nothing to report.

## Consent

Written informed consent for publication was obtained from the patient.

## Conflicts of Interest

The authors declare no conflicts of interest.

## Data Availability

The data that support the findings of this study are available from the corresponding author upon reasonable request.

## References

[1] Aslanabadi A. , Zare Z. , and Aslanabadi M. , The First Description of Acute Compartment Syndrome, by Al-Zahrawi, Journal of Bone and Joint Surgery. American Volume. (2023) 105, no. 1, e1, 10.2106/JBJS.22.00079, 35926179. 35926179

[2] von Volkmann R. , The Classic: Ischaemic Muscle Paralyses and Contractures, Clinical Orthopaedics and Related Research. (2007) 456, 20–21, 10.1097/BLO.0b013e318032561f. 17496749

[3] Livingston K. , Glotzbecker M. , Miller P. E. , Hresko M. T. , Hedequist D. , and Shore B. J. , Pediatric Nonfracture Acute Compartment Syndrome: A Review of 39 Cases, Journal of Pediatric Orthopedics. (2016) 36, no. 7, 685–690, 10.1097/BPO.0000000000000526, 26019026. 26019026

[4] Hope M. J. and McQueen M. M. , Acute Compartment Syndrome in the Absence of Fracture, Journal of Orthopaedic Trauma. (2004) 18, no. 4, 220–224, 10.1097/00005131-200404000-00005. 15087965

[5] Matava M. J. , Whitesides T. E. , Seiler J. G. , Hewan-Lowe K. , and Hutton W. C. , Determination of the Compartment Pressure Threshold of Muscle Ischemia in a Canine Model, Journal of Trauma. (1994) 37, no. 1, 50–58, 10.1097/00005373-199407000-00010. 8028059

[6] Köstler W. , Strohm P. C. , and Südkamp N. P. , Acute Compartment Syndrome of the Limb, Injury. (2005) 36, no. 8, 992–998, 10.1016/j.injury.2005.01.007. 16372396

[7] Livingston K. S. , Meehan W. P. , Hresko M. T. , Matheney T. H. , and Shore B. J. , Acute Exertional Compartment Syndrome in Young Athletes: A Descriptive Case Series and Review of the Literature, Pediatric Emergency Care. (2018) 34, no. 2, 76–80, 10.1097/PEC.0000000000000647, 27248777. 27248777

[8] Dewangan M. , Khare M. K. , Mishra S. , and Marhual J. C. , Acute Exercise-Induced Compartment Syndrome of the Leg- Don′t Miss It, Journal of Clinical and Diagnostic Research. (2016) 10, no. 2, Pd03-4, 10.7860/JCDR/2016/15779.7183, 27042521. PMC4800587 27042521

[9] Stollsteimer G. T. and Shelton W. R. , Acute Atraumatic Compartment Syndrome in an Athlete: A Case Report, Journal of Athletic Training. (1997) 32, no. 3, 248–250, 16558458. 16558458 PMC1320246

[10] Shah N. , Acute Exercise-Induced Compartment Syndrome of the Lower Leg: A Case Report, Current Sports Medicine Reports. (2009) 8, no. 6, 288–290, 10.1249/JSR.0b013e3181c22755, 19904066. 19904066

[11] Waterman B. R. , Liu J. , Newcomb R. , Schoenfeld A. J. , Orr J. D. , and Belmont P. J. , Risk Factors for Chronic Exertional Compartment Syndrome in a Physically Active Military Population, American Journal of Sports Medicine. (2013) 41, no. 11, 2545–2549, 10.1177/0363546513497922, 23911700. 23911700

[12] Fehlandt A. and Micheli L. , Acute Exertional Anterior Compartment Syndrome in an Adolescent Female, Medicine and Science in Sports and Exercise. (1995) 27, no. 1, 3–7, 10.1249/00005768-199501000-00002, 7898334. 7898334

[13] Konstantakos E. A. D. , Lateral Compartment Syndrome of the Lower Extremity in a Recreational Athlete: A Case Report, American Journal of Emergency Medicine. (2008) 26, no. 8, 973–975. 10.1016/j.ajem.2008.02.020 18926382

[14] Blandy J. P. and Fuller R. , March GANGRENE, Journal of Bone and Joint Surgery B. (1957) 39-B, no. 4, 679–693, 10.1302/0301-620X.39B4.679. 13491630

[15] Reneman R. S. , The Anterior and the Lateral Compartmental Syndrome of the Leg Due to Intensive Use of Muscles, Clinical Orthopaedics and Related Research. (1975) 113, no. 113, 69–80, 10.1097/00003086-197511000-00011, 1192677. 1192677

[16] Bowyer M. W. , Lower Extremity Fasciotomy: Indications and Technique, Current Trauma Reports. (2015) 1, no. 1, 35–44, 10.1007/s40719-014-0002-7.

[17] Cohn B. T. , Shall J. , and Berkowitz M. , Forearm Fasciotomy for Acute Compartment Syndrome: A New Technique for Delayed Primary Closure, Orthopedics. (1986) 9, no. 9, 1243–1246, 10.3928/0147-7447-19860901-15, 3763495. 3763495

[18] Berman S. S. , Schilling J. D. , McIntyre K. E. , Hunter G. C. , and Bernhard V. M. , Shoelace Technique for Delayed Primary Closure of Fasciotomies, American Journal of Surgery. (1994) 167, no. 4, 435–436, 10.1016/0002-9610(94)90130-9, 8179090. 8179090

[19] Keeling L. E. and Chang E. S. , Acute Exertional Compartment Syndrome of the Leg Following Brief Activity: A Case Report, JBJS Case Connector. (2020) 10, no. 3, e1900498, 10.2106/JBJS.CC.19.00498, 32773699. 32773699

[20] Rorabeck C. H. and Macnab I. , The Pathophysiology of the Anterior Tibial Compartmental Syndrome, Clinical Orthopaedics and Related Research. (1975) 113, 52–57, 10.1097/00003086-197511000-00008. 1192675

[21] Ruggeri E. , Pathogenesis of the Anterior Tibial Syndrome; Morphology and Vascularization of Sheath of the Anterior Tibial Muscle, Annali Italiani di Chirurgia. (1954) 31, no. 6, 448–464, 13218493. 13218493

[22] Hiramatsu K. , Yonetani Y. , Kinugasa K. , Nakamura N. , Yamamoto K. , Yoshikawa H. , and Hamada M. , Deep Peroneal Nerve Palsy With Isolated Lateral Compartment Syndrome Secondary to Peroneus Longus Tear: A Report of Two Cases and a Review of the Literature, Journal of Orthopaedics and Traumatology. (2016) 17, no. 2, 181–185, 10.1007/s10195-015-0373-8, 26362782. 26362782 PMC4882295

[23] Gloobe H. and Chain D. , Fibular Fibrous Arch–Anatomical Considerations in Fibular Tunnel Syndrome, Acta Anatomica. (2004) 85, no. 1, 84–87, 10.1159/000143983, 4713100. 4713100

[24] Ryan W. , Mahony N. , Delaney M. , O′Brien M. , and Murray P. , Relationship of the Common Peroneal Nerve and Its Branches to the Head and Neck of the Fibula, Clinical Anatomy. (2003) 16, no. 6, 501–505, 10.1002/ca.10155, 14566896. 14566896

[25] Warren J. D. , Blumbergs P. C. , and Thompson P. D. , Rhabdomyolysis: A Review, Muscle & Nerve. (2002) 25, no. 3, 332–347, 10.1002/mus.10053. 11870710

[26] Vanholder R. , Sever M. S. , Erek E. , and Lameire N. , Rhabdomyolysis, Journal of the American Society of Nephrology. (2000) 11, no. 8, 1553–1561, 10.1681/ASN.V1181553. 10906171

[27] Tsai W. H. , Huang S. T. , Liu W. C. , Chen L. W. , Yang K. C. , Hsu K. C. , Lin C. T. , and Ho Y. Y. , High Risk of Rhabdomyolysis and Acute Kidney Injury After Traumatic Limb Compartment Syndrome, Annals of Plastic Surgery. (2015) 74, no. 2 Supplement, S158–S161, 10.1097/SAP.0000000000000460, 25785380. 25785380

[28] Bhalla M. C. and Dick-Perez R. , Exercise Induced Rhabdomyolysis With Compartment Syndrome and Renal Failure, Case Reports in Emergency Medicine. (2014) 2014, 735820, 10.1155/2014/735820. 25105034 PMC4109118

[29] Asmara I. G. Y. , Pebruanto H. , and Winatha I. M. A. , Rhabdomyolysis With Compartment Syndrome-Induced Acute Kidney Injury in Resource-Limited Settings: A Case Report, Caspian Journal of Internal Medicine. (2022) 13, no. 4, 810–814, 10.22088/cjim.13.4.810, 36420323. 36420323 PMC9659830

[30] Landau M. E. , Kenney K. , Deuster P. , and Campbell W. , Exertional Rhabdomyolysis, Journal of Clinical Neuromuscular Disease. (2012) 13, no. 3, 122–136, 10.1097/CND.0b013e31822721ca. 22538307

[31] Schwaber M. J. , Liss H. P. , Steiner I. , and Brezis M. , Hazard of Sauna Use After Strenuous Exercise, Annals of Internal Medicine. (1994) 120, no. 5, 441–442, 10.7326/0003-4819-120-5-199403010-00029, 8304671. 8304671

[32] Alpers J. P. and Jones L. K. , Natural History of Exertional Rhabdomyolysis: A Population-Based Analysis, Muscle & Nerve. (2010) 42, no. 4, 487–491, 10.1002/mus.21740, 20730874. 20730874

